# Clinical and neuropsychological profile of Alzheimer’s disease in Tunisia and the impact of *APOE* gene

**DOI:** 10.3389/fgene.2026.1831886

**Published:** 2026-07-01

**Authors:** Alya Gharbi, Ikram Sghaier, Mohamed El Habibi, Youssef Abida, Amira Souissi, Amal Atrous, Meriem Zantour, Imen Kacem, Amina Gargouri Berrechid, Mouna Ben Djebara, Riadh Gouider

**Affiliations:** 1 Neurology Department, LR18SP03, Razi University Hospital, Tunis, Tunisia; 2 Faculty of Medicine of Tunis, University of Tunis El Manar, Tunis, Tunisia; 3 Clinical Investigation Center (CIC) “Neurosciences and Mental Health”, Razi University Hospital, Tunis, Tunisia; 4 Biotechnology Department, Higher Institute of Biotechnology Sidi Thabet, University of Manouba, Ariana, Tunisia

**Keywords:** Alzheimer disease, apoliprotein E, dementia, early onset, genetic, Tunisia

## Abstract

**Introduction:**

Alzheimer’s disease (AD), the leading cause of major neurocognitive disorder (MNCD) worldwide and in Tunisia, is strongly associated with Apolipoprotein E (*APOE*). In the present study our aims are to characterize the clinical profile of Tunisian patients, determine *APOE* allelic and genotypic frequencies, and asses their influence on disease phenotype.

**Methods:**

We included patients with a clinical diagnosis of probable or possible AD, who consulted in the Department of Neurology at Razi University Hospital over a span of 21 years. Demographic, clinical, and neuropsychological data were assessed. *APOE* genotyping was performed and its correlation to AD clinical features was evaluated.

**Results:**

Among 1,010 AD patients, the sex ratio was 0.62, with a mean age at onset of 68.9 ± 9.8 years. Consanguinity was reported in 30.2% of cases, and a family history of major neurocognitive disorder in 65.3%. The mean Mini-Mental State Examination (MMSE) and Frontal Assessment Battery (FAB) scores were 14.1 ± 7.7 and 6.5 ± 4.8, respectively. Overall, 47.0% of patients carried the *APOE* ε4 allele. The *APOE* ε3/ε3 genotype was the most frequent (45.94%), followed by *APOE* ε3/ε4 (40.99%). Age at onset was significantly earlier in *APOE* ε4 carriers compared to non-carriers (68.17 ± 10.05 vs. 69.53 ± 9.61 years; p = 0.027). Baseline cognitive performance was also significantly lower in *APOE* ε4 carriers, with reduced MMSE (11.65 ± 6.77 vs. 16.40 ± 7.85; p < 0.001) and FAB scores (5.46 ± 4.09 vs. 7.35 ± 5.11; p < 0.001). Neuropsychiatric assessment revealed significant associations between *APOE* ε4 carriage and visual hallucinations (p = 0.0015), aggressivity (p = 0.0022), disinhibition (p = 0.0039), and aberrant motor behavior (p = 0.0054). Finally, stratified analysis of the AD cohort according to educational attainment and *APOE* risk allele status demonstrated distinct patterns of cognitive impairment across the different subgroups.

**Discussion:**

The *APOE* ε4 allele, frequent in North African AD, appears to function not only as a genetic risk factor for AD, but also as a potential indicator, influencing disease onset and clinical severity.

## 1 Introduction

Alzheimer’s disease (AD) represents the most common cause of major neurocognitive disorder (MNCD), accounting for 50%–75% (Amiri et al., 2025). From a neuropathological perspective, AD is defined by the presence of two protein abnormalities: neurofibrillary tangles (NFTs), which are due to the intracellular accumulation of abnormally phosphorylated tubulin-associated unit (tau) protein, and extracellular deposits of beta-amyloid 1–42 peptide (Aβ-42 peptide), (Lane et al., 2018; Scheltens et al., 2021). In this context, lipid metabolism plays a critical role in AD pathology, as elevated cholesterol levels have been linked to the development of dementia in midlife (Panza et al., 2006). The apolipoproteins (Apo), especially the Apo E is a glycoprotein that is involved in the process of intracellular cholesterol utilization and is predominantly produced by astrocytes and less in microglia (Al-Ghraiybah et al., 2026). Accordingly, Apo E is the main apolipoprotein present in the central nervous system (CNS) and involved in brain vasculature regulation in both the presence and absence of neurodegeneration-related pathology (Al-Ghraiybah et al., 2026). The three common human isoforms of Apo E are E2, E3, and E4. ApoE can directly interact with Aβ proteins during the early stages of aggregation (Xia et al., 2024). While the different ApoE isoforms do not significantly alter the overall kinetics of Aβ aggregation, non-lipidated ApoE4–Aβ aggregates are cleared more slowly by glial cells, suggesting that ApoE4 may drive early Aβ aggregation and disease onset (Xia et al., 2024). Additionally, neurons exposed to ApoE4 show elevated levels of both endogenous and internalized Aβ42 compared with those exposed to ApoE3, indicating that the E4 isoform influences Aβ aggregation both extracellularly and intracellularly (Konings et al., 2023). Genetically, The *APOE* genotype is strongly associated with AD risk (Bellenguez et al., 2022). The *APOE-*ɛ4 isoform is recognized as the most significant genetic risk factor, with heterozygous carriers having a two-to threefold increased risk, and homozygous carriers a twelvefold higher risk, compared to individuals without the ɛ4 allele (Lane et al., 2018). Furthermore, *APOE*-ɛ4 may contribute to phenotypic variability in AD, affecting age at onset, initial symptoms, and predominant clinical features (Whitwell et al., 2021).

Due to population aging and the ongoing demographic transition in Tunisia, MNCD have become a major public health concern. According to the latest national data published in 2014, the prevalence of dementia rose by 24% over just over a decade, reaching approximately 4.6% among individuals aged 65 years and older in Tunisia. Within the same age group, the prevalence of AD was estimated at around 3.2%. (Hajem et al., 2014).

To the best of our knowledge, no genetic analysis has been conducted in a large, heterogeneous Tunisian cohort of AD regarding the impact of the *APOE* ɛ4 allele on AD phenotype and cognitive progression.

Therefore, the aim of our study was to characterize the clinical features of AD in Tunisia, to determine the allelic and genotypic frequencies of the *APOE* gene, and to assess its impact on the clinical phenotype.

## 2 Methods

### 2.1 Study subjects

A 21-year (2003–2024) observational, cross-sectional, and retrospective study was conducted at the Department of Neurology, Razi University Hospital. It included only patients diagnosed with MNCD per Diagnostic and Statistical Manual of Mental Disorders, fifth Edition (DSM-V) criteria (Sachdev et al., 2014) and those with clinically probable or possible AD according to National Institute of Neurological and Communicative Disorders and Stroke and Alzheimer’s Disease and Related Disorders Association (NINCDS-ADRDA) guidelines (McKhann et al., 2011).

A control cohort of 140 unrelated individuals was included in this study to serve as a healthy reference group for the genetic analyses. Participants were recruited from multiple regions across Tunisia, sharing a common ethnic background, with the support of the Laboratory of Biomedical Genomics and Oncogenetics at the Pasteur Institute in Tunis. Controls were matched to the AD cohort by age, sex, and education and were confirmed to be free of known disorders through clinical evaluation, including an initial general cognitive assessment, which served as the first indicator of cognitive impairment and was routinely applied in our selection of healthy controls. None of the participants had a family history of neurodegenerative diseases. To further minimize the likelihood of undetected genetic conditions, the majority of participants were genotyped using the Affymetrix Genome-Wide Human SNP Array 6.0, and a subset of 59 individuals underwent whole-exome sequencing. Collectively, these measures ensured that the control cohort would not confound the genetic association analyses and can be considered representative of the Tunisian population.

### 2.2 Clinical and neuropsychological assessment

All patients included in this study received neurological examinations and brain imaging (Computed Tomography or Magnetic Resonance Imaging). Demographic, clinical, and neuropsychological data were collected via standardized forms from patients and caregivers. Disease onset was defined by the age of first cognitive or psychiatric symptoms, with Alzheimer’s cases classified as early-onset (<65 years) or late-onset (≥65 years). Familial information included parental consanguinity and family history of major neurocognitive disorder. Personal medical history, including hypertension (HBP), diabetes, dyslipidemia, cardiovascular disease, stroke, and head injury, along with lifestyle factors such as smoking and alcohol consumption, were documented. We also assessed the presence of one or more of the five main symptoms during the initial consultation: memory disorders, language disorders, mood disorders, behavioral disorders, and delirium.

At the first consultation, each patient underwent a neuropsychological assessment. Il included the 30-item Mini-Mental State Examination (MMSE), which was standardized and validated for Tunisia and adjusted for age and education (Bellaj et al., 2008), to evaluate overall cognitive function. Based on the results, the severity of cognitive impairment (CI) was classified with the following cut-off scores: mild CI (30–20), moderate CI (19–10), and severe CI (<9), (Bellaj et al., 2008). The validated arab version of the Frontal Assessment Battery (FAB) was used to evaluate executive function (Jemaa et al., 2008). The Neuropsychiatric Inventory (NPI) was used to assess neuropsychiatric symptoms (NPS). We calculated both the frequency × severity score (NPI-FxS) and the caregiver distress score (NPI-D). In addition, we recorded the frequency of each NPI item for every patient, namely: delusions, hallucinations, agitation, depression, anxiety, euphoria, apathy, disinhibition, irritability, aberrant motor behavior, sleep disturbances, and appetite and eating disorders (Cummings et al., 1994).

Patients who did not meet the inclusion criteria, specifically those lacking initial cognitive assessment, imaging, or essential clinical data, were excluded from the study cohort.

### 2.3 Criteria for the classification of Alzheimer’s disease patients according to the age of onset

AD is traditionally classified into two forms based on an age-at-onset threshold: early-onset AD (EOAD) and late-onset AD (LOAD). EOAD accounts for approximately 5%–10% of cases and is characterized by symptom onset before 65 years of age, with a high estimated heritability ranging from 92% to 100%. In contrast, LOAD, which represents the majority of cases, is defined by symptom onset at or after 65 years of age. It typically presents with an initial episodic memory deficit followed by a progressive decline in other cognitive domains, and shows a more moderate heritability estimated at 60%–80% (Valdez-Gaxiola et al., 2024).

### 2.4 Genetic study

Peripheral venous blood was collected from participants into EDTA-containing tubes. Genomic DNA was extracted using the QIAamp® DNA Blood Mini Kit according to the manufacturer’s instructions (Qiagen GmbH, Hilden, Germany). *APOE* genotyping was performed using restriction fragment length polymorphism polymerase chain reaction (RFLP-PCR). Leukocyte-derived DNA was amplified by PCR using a DNA thermal cycler (Applied Biosystems™ Veriti) with oligonucleotide primers described by Hixson et al., (Hixson and Vernier, 1990).

Each PCR reaction was carried out in a final volume of 25 μL, containing 150 ng of leukocyte DNA, 20 pmol of each primer, 10% dimethyl sulfoxide (DMSO), and 0.025 U of Taq DNA polymerase. Amplification was performed over 30 cycles under the following conditions: initial denaturation at 95 °C for 35 s, primer annealing at 60 °C for 35 s, and extension at 72 °C for 35 s. PCR products were verified by electrophoresis on a 2% agarose gel.

Samples showing adequate amplification were subsequently subjected to RFLP analysis. Ten microliters of PCR product were digested overnight at 37 °C with the HhaI restriction enzyme, and the resulting fragments were separated by electrophoresis. *APOE* genotypes were determined based on characteristic fragment size patterns as follows: ɛ2/ɛ2 (91 and 83 base pair (bp)), ɛ3/ɛ3 (91 and 48 bp), ɛ4/ɛ4 (72 and 48 bp), and heterozygous genotypes ɛ2/ɛ3 (91, 83, and 48 bp), ɛ3/ɛ4 (91, 72, and 48 bp), and ɛ2/ɛ4 (91, 83, 72, and 48 bp) (Hixson and Vernier, 1990). Different genotypes found were further validated by Sanger sequencing using an Applied Biosystems 3500 Genetic Analyzer and analysis were done via Sequence Scanner Software.

### 2.5 Database and statistical analysis

We analyzed demographic and clinical characteristics, *APOE* genetic frequencies, and the relationship between clinical variables and *APOE* genotype across the cohort and its subgroups. Categorical variables were reported as counts and percentages, while continuous variables were presented as means ± standard deviation (SD) or medians with interquartile ranges, as appropriate. The AD and control groups were comparable in terms of sex distribution, age, and education level, with no statistically significant differences observed (p > 0.05), confirming appropriate matching.

To assess the association between *APOE-*ɛ4 carriage and cohort characteristics, we performed multivariable logistic regression, adjusting for sex, education, cardiovascular and metabolic comorbidities (hypertension, diabetes, dyslipidemia, cardiovascular disease, stroke), history of traumatic brain injury, smoking, and alcohol consumption. Education was subsequently included as a categorical covariate in all multivariable analyses to control for its potential confounding effect on cognitive, neuropsychological, and neuropsychiatric outcomes.

Educational attainment was treated as a categorical variable because the exact number of years of schooling was not consistently available in the clinical records; in several cases, only the educational level was documented. Therefore, according to the Tunisian educational system, AD patients were classified into four educational categories: illiterate (no formal education), primary education (1–6 years of schooling), secondary education (7–13 years of schooling), and university education (≥14 years of schooling). Adjusted effect estimates were reported as odds ratios (OR) for logistic regression analyses and standardized β coefficients for linear regression analyses, together with their corresponding 95% confidence intervals (CIn) and p-values, in order to provide both the magnitude and precision of the observed associations.

To further investigate the potential moderating effect of education, the cohort was stratified into four educational subgroups in Table 4. Within each educational subgroup, comparisons were performed between *APOE* ε4 carriers and non-carriers using separate multivariable regression models, allowing the assessment of *APOE* ε4-associated effects within each educational category independently.

To account for multiple comparisons, Bonferroni correction was applied, setting the significance threshold at p = 0.005 (0.05/11). All statistical analyses and figure generation were conducted using R version 3.6.0 (R Core Team) for Windows, with packages including, but not limited to, ggplot2, dplyr, tidyr, readr, stats, fmsb, and ggradar.

### 2.6 Ethics considerations

The study was approved by the Razi University hospital ethic committee. All subjects conformed to the principles outlined in the Declaration of Helsinki and were informed about the purposes of the study.

## 3 Results

### 3.1 Overview of the study population’s characteristics

A total of 1010 AD patients were included in this study. The total cohort showed a female predominance, with a sex ratio of 0.62. The average age at the first consultation was 72.66 ± 9.73 years. The mean diagnostic delay was 3.77 ± 3.30 years, and the average age at AD onset was 68.89 ± 9.84 years. EOAD represented 34.06% of the cohort, with a mean onset age of 51.0 ± 4.11 years. The remaining majority, diagnosed with LOAD, had a mean onset age of 74.6 ± 6.1 years.

Regarding educational level, nearly half of the participants (49.11%) were illiterate, while only 10.79% had a university education. A high prevalence of consanguinity (30.20%) and a family history of MNCD (65.25%) were observed (Table 1).

**TABLE 1:** Demographic characteristics of the study cohort.

Variables	Total cohort n = 1010	*APOE* status		
		*APOE ε*4 n = 475	*APOE* non-*ε*4 n = 535	P value
Gender (M/F), n (%)	387/623 (61.68/38.32)	183/292 (38.53/61.47)	204/331 (38.13/61.87)	0.897
Age at 1st evaluation, mean ± SD (years)	72.66 ± 9.73	72.14 ± 9.78	73.12 ± 9.67	0.110
Disease duration at 1st evaluation, mean ± SD (years)	3.77 ± 3.30	3.89 ± 3.35	3.59 ± 3.24	0.063
Age at disease onset, mean ± SD (years)	68.89 ± 9.84	68.17 ± 10.05	69.53 ± 9.61	**0.027**
Educational level, n (%)				
*Illiterate*	496 (49.11)	244 (51.37)	252 (47.10)	0.661
*Primary school*	223 (22.08)	98 (20.63)	125 (23.36)	
*High school*	157 (15.54)	69 (14.53)	88 (16.45)	
*University*	109 (10.79)	52 (10.95)	57 (10.65)	
*Not specified*	25 (2.48)	12 (2.53)	13 (2.43)	
Familial history, n (%)				
*Parental consanguinity, n (%)*	305 (30.20)	136 (28.63)	169 (31.59)	0.307
*Family history of MNCD, n (%)*	659 (65.25)	317 (66.74)	342 (63.93)	0.645
Personal medical history, n (%)				
*Hypertension*	439 (43.47)	200 (42.11)	239 (44.67)	0.623
*Diabetes*	251 (24.85)	100 (21.05)	151 (28.22)	**0.0017**
*Dyslipidemia*	157 (15.54)	65 (13.68)	92 (17.20)	0.1584
*Stroke*	53 (5.25)	26 (5.47)	27 (5.05)	0.089
*Cardiopathy*	114 (11.29)	42 (8.84)	72 (13.46)	0.067
*Head injury*	95 (9.41)	45 (9.47)	50 (9.35)	0.125
Personal habits, n (%)				
*Smoking*	290 (28.71)	142 (29.89)	148 (27.66)	0.608
*Alcohol consumption*	96 (9.50)	38 (8.00)	58 (10.84)	0.306

M, Male; F, Female; n, Number; APOE, Apolipoprotein E; SD, standard deviations; Numbers on bold are considered statistically significant (p < 0.05).

In terms of medical history, 43.47% of patients had hypertension, 24.85% had diabetes, 15.54% had dyslipidemia, 11.29% had cardiopathy, and 9.41% had a history of head injury. Stroke was the least reported risk factor, observed in 5.25% of AD cases. Smoking was the most common lifestyle factor, with 28.71% of patients being smokers (Table 1).

Clinical data analysis revealed a high prevalence of memory disorders at first consultation (95.94%), followed by mood disorders (53.86%), behavioral disorders (50.5%), and language disorders (41.68%). Delirium was reported in a smaller proportion of cases (28.61%) (Table 2).

**TABLE 2:** Clinical and neuropsychological characteristics of AD patients according to *APOE* ε4 status.

Variables	Total cohort n = 1010	*APOE* status		p value^a^	OR/β	95% CI_n_
		*APOE ε*4 n = 475	*APOE* non-*ε*4 n = 535			
Symptoms at first consultation, n (%)						
Memory disorders	969 (95.94)	461 (97.05)	508 (94.95)	0.837	0.99	0.95–1.04
Language disorders	421 (41.68)	208 (43.79)	213 (39.81)	0.877	1.01	0.91–1.12
Mood disorders	544 (53.86)	255 (53.68)	289 (54.02)	0.169	1.08	0.97–1.21
Behavioral disorders	510 (50.50)	254 (53.47)	256 (47.85)	**0.0039**	1.82	1.56–2.13
Delirium	289 (28.61)	61 (12.84)	71 (13.27)	0.027	1.04	1.02–1.18
Neuropsychological characteristics						
MMSE, mean ± SD median [IQR]	14.11 ± 7.72 14.0 [9.0–20.0]	11.65 ± 6.77 11.0 [7.0–16.0]	16.40 ± 7.85 17.0 [11.0–23.0]	**0.00014**	1.96	1.97–2.72
FAB score, mean ± SD median [IQR]	6.47 ± 4.75 5.0 [3.0–9.0]	5.46 ± 4.09 4.0 [3.0–7.0]	7.35 ± 5.11 6.0 [4.0–10.0]	**<0.0001**	1.78	1.62–2.26
Evaluation of psychiatric and behavioral symptoms						
NPI-FxS, mean ± SD median [IQR]	42.19 ± 30.66 36 [16.0–61.0]	44.93 ± 30.57 40 [19.3–62.0]	39.45 ± 30.56 31.5 [14.0–59.0]	0.839	0.99	0.89–1.11
NPI-D, mean ± SD median [IQR]	19.31 ± 13.32 17 [8.0–29.0]	19.72 ± 13.30 17 [8.0–27.0]	18.86 ± 13.37 15 [7.3–30.0]	0.837	0.96	0.81–1.05
Altered NPI items, n (%)						
Delusion	289 (28.61)	139 (29.26)	150 (28.04)	0.0128	1.07	1.04–1.14
Visual hallucinations	398 (39.41)	207 (43.58)	191 (35.70)	**0.0015**	1.72	1.46–2.02
Aggressivity	348 (34.46)	187 (39.37)	161 (30.09)	**0.0022**	1.09	1.01–1.19
Depression	381 (37.72)	185 (38.95)	196 (36.64)	0.761	1.00	0.87–1.17
Anxiety	323 (31.98)	158 (33.26)	165 (30.84)	0.385	0.93	0.83–1.04
Euphoria	110 (10.89)	68 (14.32)	42 (7.85)	0.704	0.98	0.77–1.10
Apathy	316 (31.29)	174 (36.63)	142 (26.54)	0.323	0.87	0.85–1.14
Disinhibition	289 (28.61)	152 (32.00)	137 (25.61)	**0.0039**	1.62	1.02–1.87
Irritability	459 (45.45)	235 (49.47)	224 (41.87)	0.889	1.01	0.89–1.09
Aberrant motor behavior	375 (37.13)	202 (42.53)	173 (32.34)	**0.0054**	1.90	1.12–1.92
Sleep disorders	431 (42.67)	215 (45.26)	216 (40.37)	0.0334	1.03	1.00–1.21
Appetite	276 (27.33)	145 (30.53)	131 (24.49)	0.684	1.02	0.91–1.10

N, number; APOE, Apolipoproteine E; MMSE, Mini Mental Scale Examination; FAB, Frontal Assessement Battery; IQR, Interquartile range (1^st^ quartile - 3^rd^ quartile); SD, standard deviations; NPI, neuropsychiatric Inventory; NPI-FxS, Neuropsychiatric Inventory–Frequency × Severity; NPI-D, Neuropsychiatric Inventory–Distress; Numbers on bold are considered statistically significant (p < 0.005).

^a^ adjusted according to sex, education, cardiovascular and metabolic comorbidities (hypertension, diabetes, dyslipidemia, cardiovascular disease, stroke), traumatic brain injury, smoking, and alcohol consumption.

Mean MMSE score was 14.11 ± 7.72, while the mean FAB score was 6.47 ± 4.75. Regarding NPS, high scores on the NPI were noted, with a mean NPI-FxS of 42.19 ± 30.66 and a mean NPI-D of 19.31 ± 13.32. An analysis of the NPI items revealed that the most frequently impaired domains in AD patients were irritability (45.45%), sleep disturbances (42.67%), visual hallucinations (39.41%), and depression (37.72%). Other items were less commonly reported, with euphoria being the least observed (10.89%) (Table 2).

### 3.2 Association of APOE genotypes and AD

**FIGURE 1:**
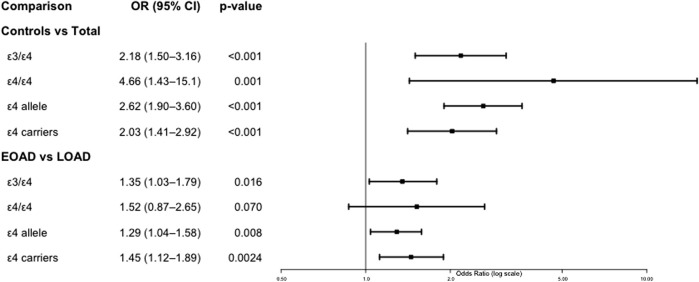
Forest plot showing the association of *APOE* genotypes with Alzheimer’s disease risk in patients versus healthy controls, with additional stratification of the AD cohort according to age at onset (EOAD and LOAD).

The genetic study was performed in all patients. The *APOEε*4 allele carriers represented 47.03% of the AD cohort. The *APOE* ɛ3ɛ3 genotype was the most frequent (45.94%) in the total AD population followed by *APOE* ɛ3ɛ4 (40.99%). The *APOE ε*4*ε*4 genotype was found in 5.84% of cases. A significant difference in genotype distribution was observed between AD cases and controls. Specifically, the frequencies of both heterozygous and homozygous *APOE* ɛ4 genotypes were notably higher in AD patients compared to controls. Indeed, *APOE* ɛ3ɛ4 represented 40.99% of AD vs. 28.48% (p < 0.001) of controls and *APOE* ɛ4ɛ4 represented 5.84% of AD vs. 1.89% of controls (p = 0.001). Our analysis indicated that individuals carrying at least one ɛ4 allele have a 2.2-fold increased risk of developing AD compared to non-carriers. This risk rises to 4.7-fold in individuals carrying two copies of the ɛ4 allele (Table 3; Figure 1).

**TABLE 3:** *APOE* distribution among total AD cohort and stratified according to age of disease onset (EOAD and LOAD).

*APOE*	Total cohort n = 1010	Controls n = 158	P^1^ value	OR^1^ (95% CI_n_)	EOAD n = 344	LOAD n = 665	P^2^ value^a^	OR^2^ (95% CI_n_)
Genotype, n (%)								
ɛ3 ɛ3	464 (45.94)	110 (69.62)	—	Ref (1.00)	144 (41.86)	320 (48.12)	—	Ref. (1.00)
ɛ2 ɛ4	2 (0.19)	00 (0.00)	—	—	2 (0.58)	00 (0.00)	—	—
ɛ2 ɛ3	71 (7.03)	00 (0.00)	—	—	17 (4.94)	54 (8.12)	0.145	0.91 (0.81–1.03)
ɛ3 ɛ4	414 (40.99)	45 (28.48)	**<0.001**	2.18 (1.50–3.16)	157 (45.63)	257 (38.64)	**0.0039**	1.20 (1.13–1.29)
ɛ4 ɛ4	59 (5.84)	03 (1.89)	**0.001**	4.66 (1.43–15.1)	24 (6.97)	35 (5.26)	0.058	1.14 (1.02–1.19)
*Allele, n (%)*								
ɛ3	1413 (69.95)	265 (83.86)	—	Ref (1.00)	462 (67.15)	951 (71.50)	—	Ref (1.00)
ɛ2	73 (3.61)	00 (0.00)	**<0.001**	0.79 (0.77–0.82)	19 (2.76)	54 (4.06)	0.329	0.95 (0.85–1.05)
ɛ4	534 (26.44)	51 (16.14)	**<0.001**	2.62 (1.90–3.60)	205 (29.79)	327 (24.58)	**<0.001**	1.12 (1.10–1.25)
*Genotype analysis according to* ɛ4 carriage, *n (%)*								
*APOE*ɛ4	475 (47.03)	48 (30.37)	**<0.001**	2.03 (1.41–2.92)	183 (53.19)	292 (43.90)	**0.0019**	1.51 (1.13–1.98)
Non-*APOE*ɛ4	535 (52.97)	110 (69.62)			161 (46.80)	374 (56.24)		

N, number; AD, Alzheimer’s disease; LOAD, Late Onset Alzheimer’s disease; EOAD, early onset alzheimer disease; APOE, Apolipoprotein E; APOEɛ4, carriers of APOE ɛ4 (including ɛ3/ɛ4, ɛ4/ɛ3 and ɛ4/ɛ2) Non-APOE ɛ4: non-carriers of APOE ɛ4 (including ɛ3/ɛ3 and ɛ2/ɛ3); P^1^, Comparison between AD, patients and controls matched in terms of sex, age, and education level; OR^1^, odds ratio of P1; P^2^ value: comparison between EOAD, and LOAD; OR^2^, odds ratio of P2, CI, confidence interval in 95%.

^a^ Number on bold was considered significant (p < 0.005) after the Bonferroni correction for sex, education, cardiovascular and metabolic comorbidities (hypertension, diabetes, dyslipidemia, cardiovascular disease, stroke), traumatic brain injury, smoking, and alcohol consumption.

Upon analyzing the distribution of *APOE* alleles in our AD cohort, significant differences were observed in the frequency of the *APOE* ɛ4 allele compared to the control group, regardless of the number of allele copies. Moreover, 47.03% (475/1010) of AD patients were carriers of the ɛ4 allele, compared to only 3.03% (48/158) of the controls (p < 0.001) (Table 3; Figure 1).

### 3.3 *APOE* genotype and its impact on AD clinical features

When comparing the demographic, familial, and clinical characteristics of AD patients based on *APOE* carrier status, we observed several significant differences (Tables 1 and 2).

**FIGURE 2:**
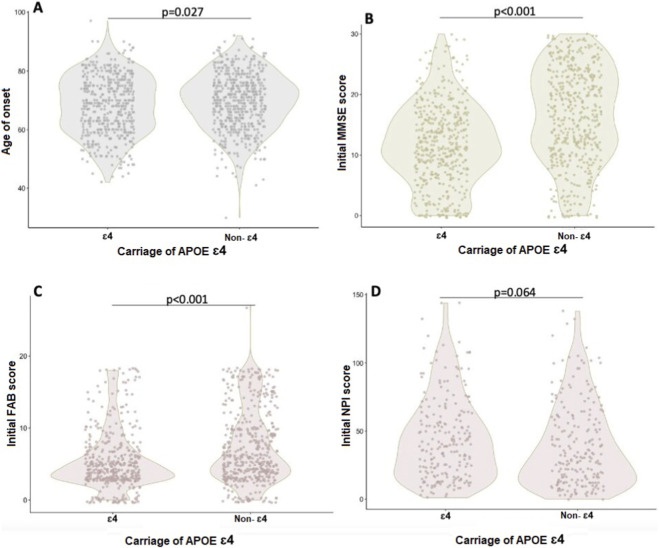
Violin plots illustrating differences in clinical characteristics according to *APOE* ε4 carrier status **(A)** comparison of age at disease onset between *APOE* ε4 carriers and non-carriers; **(B)** comparison of initial Mini-Mental State Examination (MMSE) scores according to *APOE* ε4 carriage; **(C)** differences in initial Frontal Assessment Battery (FAB) scores between *APOE* ε4 carriers and non-carriers; **(D)** comparison of initial Neuropsychiatric Inventory (NPI) scores based on *APOE* ε4 carrier status.

Although sex distribution and educational level did not differ significantly between groups, the age at onset was significantly lower in *APOE* ε4 carriers (68.17 ± 10.05 years) than in non-carriers (69.53 ± 9.61 years) (p = 0.027) (Table 1; Figure 2A). Furthermore, the comparison of genotypic and allelic frequency distributions between EOAD and LOAD revealed statistically significant differences after adequate adjustment. Indeed, the ε3ε4 genotype was significantly more frequent in EOAD compared to LOAD (45.63% vs. 38.64%, respectively; *p* = 0.0039). Similarly, *APOE* ε4 carriers were also more frequent among EOAD patients (53.19%) than among LOAD patients (43.90%) (p = 0.0019) (Table 3; Figure 1).

When comparing personal medical risk factors according to *APOE* status, significant differences were found for diabetes (p = 0.0017), with a higher prevalence in non-ɛ4 carriers (28.22% vs. 21.05%).

Analysis of the impact of *APOE* ε4 status on the frequency of inaugural symptoms revealed no significant differences between groups, with the exception of behavioral disorders, which were significantly more prevalent among ε4 carriers (p = 0.0039).

Baseline cognitive performance was significantly poorer in *APOE* ε4 carriers compared to non-carriers, as reflected by lower MMSE scores (11.65 ± 6.77 vs. 16.40 ± 7.85; p < 0.001) (Figure 2B; Table 2). Consistently, FAB scores at baseline were also reduced in ε4 carriers (5.46 ± 4.09 vs. 7.35 ± 5.11; p < 0.001) (Table 2; Figure 2C).

**FIGURE 3:**
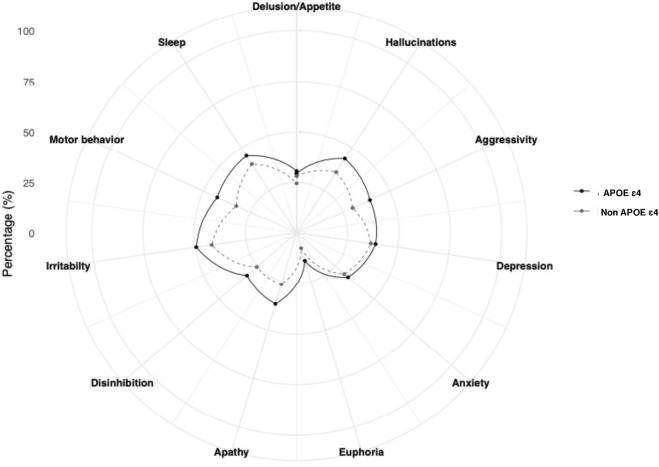
Comparison of altered neuropsychiatric items in AD patients according to *APOE* ε4 carriage.

Regarding NPI scores, total NPI-D values were comparable between groups (p = 0.837). Likewise, no significant difference was observed in NPI-F × S scores between *APOE* ε4 carriers and non-carriers (44.93 vs. 39.45; p = 0.839) (Figure 2D). Stratified analysis of NPI items revealed significant associations between the presence of the *APOE* ɛ4 allele and the development of visual hallucinations (p = 0.0015), aggressivity (p = 0.0022), disinhibition (p = 0.0039), and aberrant motor behavior (p = 0.0054), with higher frequencies of these symptoms among *APOE* ɛ4 carriers (Table 2; Figure 3).

Given that educational attainment represents a critical factor in cognitive assessment, we stratified our cohort into four subgroups according to the Tunisian educational system (illiterate, primary, secondary, and university levels). This approach aimed to determine whether the impact of the *APOE* ε4 allele differs across educational strata, and to identify domain-specific cognitive alterations that may be evident within particular subgroups but obscured in analyses conducted on the overall cohort (Table 4).

**TABLE 4:** Clinical and neuropsychological characteristics of AD patients according to *APOE* ε4 status stratified according to educational level.

Variables	Educational level^a^ ^,^ ^b^							
	Illiterate		Primary		Secondary		University	
	P value	OR/β (95% CI_n_)	P value	OR/β (95% CI_n_)	P value	OR/β (95% CI_n_)	P value	OR/β (95% CI_n_)
Symptoms at first consultation								
Memory disorders	0.172	1.22 (0.91-1.63)	0.215	1.59 (0.75-3.34)	0.867	0.96 (0.64-1.46)	0.537	1.21 (0.64-2.28)
Language disorders	0.413	0.96 (0.87-1.05)	**0.00015**	1.31 (1.14-1.50)	0.524	1.05 (0.89-1.25)	0.482	1.08 (0.86-1.36)
Mood disorders	0.343	0.95 (0.86-1.05)	**0.00016**	1.20 (1.04-1.38)	0.303	0.91 (0.71-1.16)	0.760	1.04 (0.82-1.29)
Behavioral disorders	0.185	1.03 (0.48-1.12)	0.908	0.99 (0.86-1.13)	**0.0018**	1.22 (1.03-1.45)	0.565	1.06 (0.85-1.33)
Delirium	0.475	0.95 (0.83-1.08)	0.499	1.07 (0.86-1.35)	0.945	1.09 (0.92-1.29)	0.905	1.03 (0.66-1.61)
Neuropsychological characteristics								
Mean initial MMSE score	**0.0019**	1.98 (1.71-2.13)	**2.07e** ^**-14**^	1.96 (1.79-2.25)	**9.18e** ^**-08**^	1.97 (1.96-1.98)	**0.00101**	1.98 (1.96-1.99)
Mean Initial FAB score	0.082	0.98 (0.97-1.00)	**0.00036**	1.97 (1.95-1.98)	**0.00461**	1.81 (1.66-1.99)	**0.00849**	2.13 (1.67-2.73)
Evaluation of psychiatric and behavioral symptoms								
Mean NPI-FxS score	0.222	1.00 (0.99-1.03)	0.163	1.02 (0.77-1.35)	0.0918	1.00 (0.99-1.00)	0.958	0.99 (0.99-1.01)
Mean NPI-D score	0.939	1.01 (0.85-1.19)	0.398	1.05 (0.99-1.17)	0.195	1.01 (0.99-1.02)	0.373	0.98 (0.97-1.20)
Altered NPI items								
Delusion	0.546	1.04 (0.91-1.17)	0.761	0.96 (0.79-1.18)	0.0516	1.26 (1.00-1.59)	0.133	0.79 (0.59-1.07)
Visual hallucinations	0.144	0.93 (0.84-1.03)	**0.007**	1.22 (1.05-1.42)	**0.00145**	1.36 (1.12-1.65)	**0.00301**	1.30 (1.02-1.65)
Aggressivity	0.867	1.00 (0.90-1.13)	0.0152	1.13 (1.01-1.34)	**0.00278**	1.29 (1.04-1.61)	**0.0013**	1.65 (1.32-2.06)
Depression	0.346	1.07 (0.92-1.23)	0.808	0.97 (0.78-1.20)	0.656	1.04 (0.79-1.37)	0.665	1.08 (0.76-1.88)
Anxiety	0.696	1.03 (0.89-1.17)	**0.0020**	1.77 (1.62-1.95)	**0.00713**	1.43 (1.11-1.85)	0.723	0.93 (0.62-1.39)
Euphoria	0.387	1.06 (0.92-1.23)	0.056	1.26 (0.99-1.60)	0.176	1.28 (0.90-1.62)	0.097	1.37 (0.91-2.07)
Apathy	0.181	1.08 (0.96-1.21)	0.080	0.70 (0.47-1.05)	0.418	1.09 (0.87-1.37)	0.605	1.07 (0.81-1.41)
Disinhibition	0.694	1.02 (0.91-1.14)	0.255	1.12 (0.92-1.34)	0.095	1.21 (0.96-1.53)	0.792	0.95 (0.72-1.27)
Irritability	0.910	1.00 (0.87-1.15)	0.307	1.10 (0.90-1.35)	0.515	0.92 (0.71-1.19)	0.185	1.22 (0.90-1.66)
Aberrant motor behavior	**0.0024**	1.62 (1.27-1.83)	**0.0016**	1.51 (1.26-1.67)	0.383	1.10 (0.88-1.38)	0.211	1.20 (0.91-1.61)
Sleep disorders	0.934	0.99 (0.88-1.12)	0.283	1.09 (0.91-1.31)	0.730	0.96 (0.77-1.20)	0.895	0.97 (0.73-1.30)
Appetite	0.599	1.03 (0.91-1.15)	0.544	1.06 (0.87-1.30)	0.064	1.26 (0.99-1.62)	0.673	0.94 (0.70-1.25)

MMSE, mini mental scale examination; FAB, frontal assessment battery; NPI-FxS, Neuropsychiatric Inventory Frequency × Severity score; NPI-D, Neuropsychiatric Inventory–Distress score. OR, Odds Ratio. CI, confidence interval, Numbers on bold are considered statistically significant (p < 0.005 (0.05/10). Primary: participants with 1–6 years of formal schooling. Secondary: participants with 7–13 years of schooling. University: participants with 14 or more years of education.

^a^ Within each subgroup, comparisons were conducted between APOE-ε4, carriers and non-carriers.

^b^ Education levels were categorized into groups and stratified according to the national education system in Tunisia.

Among illiterate patients, carriage of the *APOE* ε4 allele was significantly associated with lower baseline MMSE scores (p = 0.0019). In this subgroup, aberrant motor behavior was the only cognitive/behavioral domain showing a significant deficit linked to *APOE* ε4 status (p = 0.0024).

In AD patients with a primary educational level, *APOE* ε4 carriers exhibited lower baseline MMSE and FAB scores. Moreover, clinical features at first consultation—particularly language impairment and mood disturbances—were significantly more frequent among *APOE* ε4 carriers (p = 0.00015 and p = 0.00016, respectively).

Focusing on AD patients with a secondary level of education, we observed that behavioral disturbances were more frequently reported as presenting symptoms among *APOE* ε4 carriers (p = 0.0018). In this subgroup, baseline MMSE and FAB scores were also significantly lower in individuals carrying the risk allele (p < 0.001 and p = 0.004, respectively). Furthermore, analysis of NPI domains revealed that hallucinations were significantly associated with *APOE* ε4 carriage (p = 0.0014) as well as aggressivity (p = 0.0027). Finally, in AD patients with the highest educational level, the mean baseline MMSE score remained significantly lower among *APOE*ε4 carriers (p = 0.001). Regarding neuropsychiatric symptoms, visual hallucinations were significantly associated with *APOE* ε4 carriage in this subgroup (p = 0.003) and aggressivity (p = 0.0027).

## 4 Discussion

In the present study, we analyzed the largest North African cohort of AD cases genotyped for *APOE* reported to data (Rassas et al., 2012; Ramadan et al., 2019; Emrani et al., 2020; Xu et al., 2021). To our knowledge, no previous investigation has comprehensively characterized the clinical spectrum of AD in relation to its key determinants in Tunisia or across North Africa. Existing literature from the region remains limited, with only recent study addressing survival and preventive strategies with the broader Middle East and North Africa (MENA) region (Amiri et al., 2025).

Regarding demographic characteristics, our cohort demonstrated a female predominance (sex ratio = 0.62), consistent with prior epidemiological findings (Amiri et al., 2025). This observation is further supported by experimental evidence, as murine hippocampal expression studies have shown that genes linked to energy metabolism and amyloid deposition are altered earlier in females, potentially increasing their susceptibility to developing the disease (Zhao et al., 2016).

The average age at onset of AD in our cohort was 68.89 ± 9.84 years, with 34.06% of cases classified as EOAD. Although EOAD is typically reported to account for approximately 5%–6% of AD cases (Zhu et al., 2015; Mendez, 2019; Lin et al., 2025), its prevalence varies across populations. In our cohort, the relatively high proportion of EOAD may reflect population-specific genetic influences. We hypothesize that this increased frequency could be partly attributable to genetic drift, potentially involving recessive inheritance patterns or oligogenic mechanisms. This interpretation is supported by the high rate of consanguinity in the Tunisian population (30.2%), and the substantial proportion of individuals with a family history of MNCD reaching (65.3%) in our cohort. These findings are consistent with previous studies suggesting that recent consanguinity and increased autozygosity, even in otherwise outbred populations, are associated with an elevated risk of developing AD (Napolioni et al., 2021). Furthermore, the monocentric design of our study may have introduced a selection bias, potentially resulting in an overrepresentation of specific clinical phenotypes, particularly EOAD.

The mean diagnostic delay in our cohort was 3.8 years, exceeding estimates reported in the literature. In European settings, the average time to dementia diagnosis has been reported to range between 1.6 and 2.6 years (Dumas et al., 2023). The longer delay observed in our population may reflect limitations in healthcare resources and workforce capacity. In the context of a projected increase in the number of individuals living with AD, these constraints pose substantial challenges to the healthcare system and highlight the need for improved diagnostic pathways, sustainable care models, and strategic health system planning (Power et al., 2023).

Regarding educational background, nearly half of the patients (49.11%) were illiterate (49.11%), supporting previous findings that AD prevalence is higher among individuals with limited or no formal education. This association may reflect the influence of illiteracy on cognitive performance, particularly in domains such as memory and visuoconstructive skills (Youn et al., 2011).

In our cohort, hypertension was the most prevalent comorbidity among AD patients (43.47%), followed by diabetes (24.85%) and dyslipidemia (15.54%). Comorbid conditions in AD may act either risk factors for the disease development or as a consequence of its pathological processes, ultimately contributing disease progression (Maciejewska et al., 2021). Chronic hypertension, for instance, promotes intracranial and extracranial atherosclerosis, resulting in lacunar infarcts, white matter lesions, and brain atrophy (Yan et al., 2024). These vascular changes can increase the metabolism of amyloid precursor protein (APP) and impair the clearance of Aβ, thereby promoting AD pathology. Diabetes is also recognized as a major risk factor for AD, as insulin plays a critical role in neuronal protection, synaptic function regulation, and cognitive processes in the brain (Chornenkyy et al., 2019; Maciejewska et al., 2021). Insulin may additionally influence Aβ production and degradation by modulating enzyme activity or enhancing peptide synthesis (Yang et al., 2013). Finally, dyslipidemia, characterized by elevated total cholesterol (TC) and alterations in low-density lipoprotein cholesterol (LDL-C), has likewise been associated with AD. Recent meta-analyses indicate that individuals with AD often exhibit higher serum TC and LDL-C levels compared to cognitively normal individuals, supporting the link between lipid metabolism and AD pathogenesis (Liu et al., 2020; Maciejewska et al., 2021; Yue et al., 2025).

A key finding of this study concerns the distribution of *APOE* genotypes and alleles in AD and their comparison with healthy controls in a North African population, specifically Tunisia. We observed that carriers of the *APOE ε*4 allele accounted for 47.03% of the AD cohort, consistent with previously reported data. This result aligns with a large meta-analysis including 142 independent samples from Asian, European and American countries, which reported a pooled prevalence of *APOE ε*4 carriers in AD patients of approximately 48.7% (Ward et al., 2012). Within our cohort, the frequency of the *APOE ε*3/*ε*4 genotype was 40.99%, and that of the *APOE ε*4/*ε*4 genotype accounted for 5.84%, both exceeding the corresponding frequencies in healthy controls. Across 73 studies, the average frequency of the *ε*4/*ε*4 genotype in AD patients was 9.6%, with the highest prevalence observed in Northern Europe (14.1%) and the lowest in Asia (7.7%) and Southern Europe (4.6%). Accordingly, the prevalence of *ε*4 homozygotes in our cohort resembles that reported in Southern European populations, a similarity that may reflect historical gene flow and shared genetic backgrounds across the Mediterranean basin (Ward et al., 2012).

We next examined the influence of *APOE* genotype on demographic and clinical characteristics of AD in Tunisian patients. Carriage of the *APOE*ε4 allele was significantly associated with earlier disease onset (p = 0.027), with ε4 carriers developing AD approximately 1.3 years earlier than non-carriers. While this difference is modest from a clinical standpoint, it remains statistically robust and offers important insights at the population level, emphasizing the modulatory effect of *APOE*ε4 on disease timing and its potential utility in risk stratification frameworks. Moreover, the frequency of *APOE*ε4 differed significantly between LOAD and EOAD subgroups (43.9% vs. 53.2%, p = 0.0019). Carriers of the APOE ε4 allele were approximately 1.5 times more likely to develop Alzheimer’s disease at an earlier age compared to non-carriers, highlighting the role of this allele in phenotypic heterogeneity and in accelerating disease onset. Overall, these results emphasize the influence of *APOE*ε4 on both the age of onset and the clinical expression of Alzheimer’s disease, supporting its importance as a major genetic risk factor in the Tunisian population. Our findings are consistent with previous studies reporting that the presence of at least one *APOE*ε4 allele is associated with earlier disease onset (Montufar et al., 2017; Palmer et al., 2023; Polsinelli et al., 2023).

In addition, the relatively high proportion of early-onset Alzheimer’s disease cases in our cohort may be partly explained by the high rate of consanguinity in North Africa, particularly in Tunisia. Consanguinity, estimated to range from 20% to 40% in this region compared to 0%–10% worldwide, increases the genetic burden and may contribute to earlier disease manifestation (Mezzi et al., 2024), (Inbreeding by Country, 2026).

Pathophysiologically, this association is attributed to the co-deposition of ApoE and Aβ in amyloid plaques, a critical mechanism in AD pathogenesis (Namba et al., 1991).

Experimental studies in amyloid model mice have shown that the knockout of endogenous ApoE alters the Aβ plaques morphology, highlighting its essential role in Aβ fibrillogenesis and deposition. Importantly, this effect is isoform-dependent: ApoE4 is associated with increased Aβ accumulation in brain tissue, accelerating the onset of AD symptoms (Bales et al., 2009; Mahan et al., 2022).

Significant differences in comorbidities were observed, most notably the prevalence of diabetes (p = 0.0017), with non-carriers of the *APOE*ε4 allele exhibiting a higher rate of diabetes compared to carriers (28.2% vs. 21.0%). Although the relationship between *APOE* and diabetes has been extensively studied, findings remain inconsistent, and only a limited number of investigations have specifically addressed the interaction between *APOE* variants, diabetes, and cognitive function in aging populations (Lee et al., 2022; Mateo-Gallego et al., 2023).

Beyond comorbidities, our study identified marked phenotypic variability according to *APOE* status, particularly in cognitive profiles and neuropsychiatric manifestations. ε4 carriers demonstrated significantly poorer cognitive performance, reflected in lower MMSE (11.65 vs. 16.40) and FAB (5.46 vs. 7.35) scores. These findings are consistent with prior reports showing that AD patients carrying the *APOE* ε4 allele exhibit more pronounced memory deficit compared to non-carriers (Kim et al., 2018; Weintraub et al., 2020). Furthermore, age-related memory decline in cognitively healthy individuals has also been shown to differ according to *APOE* genotype, with ε4 carriers experiencing faster deterioration than non-carriers (Caselli et al., 2009).

This accelerated cognitive impairment is likely mediated by the role of the *APOE* ε4 allele in promoting cerebral amyloid-β accumulation, a central pathological hallmark of AD. Additionally, the association between ε4 carriage and impaired executive function is supported by neuroimaging studies, which report decreased metabolic activity in posterior brain regions and concomitant increases in anterior frontal metabolism among ε4 carriers (Gharbi-Meliani et al., 2021).

Another key finding of our study is the multifaceted nature of cognitive deficits and NPS in AD, with specific clinical profiles varying according to *APOE* genotype. Notably, *APOE* ε4 carriers demonstrated a significantly increased frequency of NPS, including visual hallucinations (p = 0.0015), aggressivity (p = 0.0022), disinhibition (p = 0.0039), and aberrant motor behavior (p = 0.0054), highlighting the allele’s contribution to more severe behavioral manifestations in AD.

Given the multifaceted role of *APOE*’s in amyloid metabolism, mitochondrial function, immune modulation, and neuronal repair, specific ApoE isoforms may contribute to an increased prevalence of NPS in AD. Consistent with this hypothesis, recent reviews have highlighted have highlighted associations between psychosis, primarily manifesting as delusions and hallucinations, and *APOE* genotypes (Lozupone et al., 2026). Furthermore, interactions between psychotic symptoms and *APOE* allele status underscore the interplay between genetic predisposition and NPS during the prodromal stages of AD (Creese et al., 2023). Emerging evidence also links psychotic manifestations and *APOE* genotypes to neuropathological changes in the temporal lobe, with *APOE* ε4 carriage associated with degeneration of specific brain regions that modulate both the occurrence and severity of psychotic symptoms (Lozupone et al., 2026) and aggressivity (Craig et al., 2004). Further research is needed to fully elucidate the mechanisms underlying the relationship between specific NPS and *APOE* genotype (Valero et al., 2020; Yoro-Zohoun et al., 2021; Berardino et al., 2025).

In the stratified analysis of Alzheimer’s disease patients according to educational level within the Tunisian system, educational attainment appeared to modulate the effect of *APOE*, as the frequency of presenting symptoms at first consultation varied across educational subgroups. A comparable pattern was observed for altered NPI domains and specific items, suggesting that educational level may influence the clinical expression of the *APOE* ε4 effect (Xiang et al., 2021). However, it is noteworthy that the initial MMSE score, reflecting global cognitive status, was consistently reduced across all subgroups regardless of educational attainment.

These findings are consistent with previous work by Langella et al. which, in a genetically characterized cohort of AD patients, reported that the impact of *APOE* ε4 varies according to educational attainment, independent of genetic profile (Langella et al., 2023). More recent studies further support a significant interaction between *APOE* ε4 carriage and education level in shaping cognitive decline trajectories in AD patients (Walsemann et al., 2025). The underlying biological mechanisms of this *APOE*–education interaction remain to be fully elucidated; however, prior evidence suggests that higher educational attainment is associated with lower levels of amyloid burden in AD patients (Gonneaud et al., 2020). Furthermore, This interaction can be understood through the relationship between cognition, brain pathology, and neuropsychiatric symptoms that recent study suggested (Goldberg et al., 2024). Studies have shown that NPI symptoms are partly driven by cognitive decline, meaning that worsening cognition may contribute to or intensify abnormal behaviors. In this context, education can be seen as a proxy for cognitive reserve, influencing the point at which underlying brain pathology begins to produce clinical neuropsychiatric symptoms. In addition, specific neuropathological features—such as neuritic amyloid plaques and Braak stage—are more strongly associated with these symptoms than diffuse amyloid deposition. This suggests that synaptic dysfunction and disruption of brain networks, rather than amyloid accumulation alone, are key mechanisms underlying complex behavioral symptoms, including psychosis.

Finally, *APOE* ε4 does not affect all cognitive domains in the same way. Its impact appears stronger on specific functions, particularly verbal episodic memory, while its association with overall global cognition is less consistent. Overall, these findings support a model in which *APOE* ε4 contributes to both cognitive decline and neuropsychiatric symptoms through partly different pathways, while education and cognitive reserve help shape how these effects are expressed clinically.

Several limitations should be acknowledged when interpreting the present findings. First, the monocentric design may have introduced a selection bias, potentially leading to an overrepresentation of specific clinical or genetic characteristics, including early-onset AD cases. Nevertheless, the study was conducted at the only public specialized Alzheimer’s disease center in Tunisia, integrated within the neurology department and functioning as a national referral center with an extended regional role across North Africa, thereby supporting the representativeness and diversity of the recruited cohort. Second, the study extended over a period exceeding 20 years, which may have introduced variability in neuropsychological and neuropsychiatric assessments due to the involvement of different neuropsychologists over time. However, all evaluations were performed using standardized and routinely applied assessment protocols, which helped reduce inter-examiner variability and ensured the comparability and consistency of cognitive and behavioral measures throughout the study period. Third, the genetic investigation was restricted to *APOE* genotyping. Other genetic variants potentially interacting with or modulating *APOE-*related effects were not explored and may have contributed to the observed phenotypic heterogeneity. Future studies integrating broader genomic approaches are therefore needed to better characterize the complex genetic architecture of AD within the Tunisian population. Finally, the relatively low frequency of the *APOE* ε4 allele observed among healthy controls (3.03%) may have amplified the estimated effect size associated with the risk allele. However, this distribution is consistent with previously published Tunisian data reporting an *APOE* ε4 frequency of approximately 2.8% in healthy individuals (Rassas et al., 2012), suggesting that our findings remain within the expected epidemiological range for this population.

To conclude, this is the largest study conducted in North Africa examining the clinical and genetic features of AD. The high prevalence of the *APOE ε4* allele in our Tunisian AD population is consistent with previous research, confirming that this allele may represent a risk factor for AD, even in Arab and African populations. Furthermore, our findings suggest that patients carrying the *APOE ε4* allele—whether heterozygous or homozygous—may exhibit a distinct and potentially more severe phenotype. Indeed, *APOE ε4* carriers not only presented with an earlier age of onset but also showed poorer cognitive performance, as reflected by lower MMSE and FAB scores, indicating greater cognitive impairment and executive dysfunction.

## 5 Conclusion

Our study sheds light on specific NPS associated with *APOE* genotype, highlighting the broader phenotypic variability linked to this genetic factor. The *APOE* ε4 allele thus appears to function not only as a genetic risk factor for AD, but also as a potential modulator influencing disease onset and clinical severity. Therefore, future research should aim to unravel the exact mechanisms through which the *APOE* locus influences the AD phenotype, investigate the potential mediating role of cognitive function, and evaluate the efficacy of therapeutic strategies targeting *APOE* modulation.

## Data Availability

The original contributions presented in the study are included in the article/supplementary material, further inquiries can be directed to the corresponding author.
